# Community-Based Event Detection in Temporal Networks

**DOI:** 10.1038/s41598-019-40137-0

**Published:** 2019-03-13

**Authors:** Pablo Moriano, Jorge Finke, Yong-Yeol Ahn

**Affiliations:** 1 Center for Complex Networks and Systems Research, School of Informatics, Computing, and Engineering, Indiana University, Bloomington, IN 47408 USA; 2 Center for Security and Privacy in Informatics, Computing, and Engineering, School of Informatics, Computing, and Engineering, Indiana University, Bloomington, IN 47408 USA; 3 Department of Electrical Engineering and Computer Science, Pontificia Universidad Javeriana, Cali, Colombia

## Abstract

We propose a method for detecting large events based on the structure of temporal communication networks. Our method is motivated by findings that viral information spreading has distinct diffusion patterns with respect to community structure. Namely, we hypothesize that global events trigger viral information cascades that easily cross community boundaries and can thus be detected by monitoring intra- and inter-community communications. By comparing the amount of communication within and across communities, we show that it is possible to detect events, even when they do not trigger a significantly larger communication volume. We demonstrate the effectiveness of our method using two examples—the email communication network of Enron and the Twitter communication network during the Boston Marathon bombing.

## Introduction

Event detection is of crucial importance in many socio-technical systems because events often bear anomalous outcomes of societal interest^1^, which range from unauthorized activities in computer networks^2^, fraudulent credit card transactions^3^ and disease outbreaks^4^. Most events of interest occur in networked systems, such as an organization, the society, or the Internet. Therefore identifying events in temporal networks has attracted much attention^5^. A key challenge in event detection is distinguishing events from natural system variations. Consider the case of email exchanges in an organization. An unusual volume of emails may not necessarily represent an event, but reflect seasonal behaviors. Communication traffic tends to vary based on particular dates (e.g., due to upcoming releases). Such variations represent a regular pattern of the email communication network and should not be associated to events^6^.

Traditional event detection methods focus on identifying changes in structural features at the macro- and microscopic level (e.g., in the distribution of the degrees of all nodes or in node properties like centrality measures)^7–14^. Model-based approaches combine block models with Bayesian change point detection^15,16^. More recent approaches analyze meso-scopic properties shared by nodes that are grouped into densely connected communities^15–17^. Simple approaches detect communities at particular time slices evaluate whether significant changes in the community structure at subsequent slices take place^17^. A key advantage of community-based methods is the robustness to fluctuations in link density^18,19^.

Here, instead of monitoring changes in the community structure itself, we propose to examine the difference between the ratio of inter- and the intra-community communication, supported by a previous finding that link information diffusion patterns with respect to communities to virality of the information^20^. The proposed method is likely to be less computationally expensive compared to other community-based methods since it does not require computing the similarity between communities of two networks every time slice.

**Figure 1:**
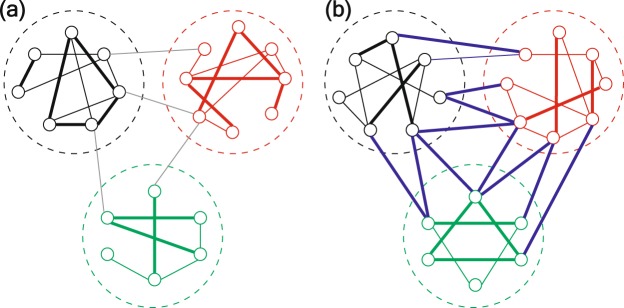
Schematic representation of the proposed event detection method. For both networks nodes are associated to the same communities but different patterns of communication within and across communities emerge. (**a**) When there is no event, most communication takes place within communities. (**b**) When a large event occurs, more communication takes place across communities because of the global relevance and the virality of the event.

Figure 1 illustrates the main idea of the method. When there is no global event, communication between nodes takes place mostly within each community (as in Fig. 1(a)). However, when a global event occurs, it spreads virally, crossing community boundaries and producing more inter-community communication (as illustrated in Fig. 1(b)). The proposed method detects such global events by monitoring the communication volume within and across communities. We demonstrate the effectiveness of the method by analyzing the email communication network of Enron (based on events reported in previous studies^21,22^) and the interactions between Twitter users during the Boston Marathon bombing.

It has been shown that many types of information in society spread like complex contagions, i.e., successful transmission depends upon interaction with multiple carriers^23^. However, as a previous study demonstrates^20^, events of global interest tend to propagate as simple contagions, where the impact of reinforcement is weak. As a result, viral information cross community boundaries easily. We build on this observation by hypothesizing that systematic major events trigger large viral cascades. For the two case studies, the email communication network and the Twitter network, the related content about the events becomes easily accessible to multiple communities.

## Results

### Enron

**Figure 2:**
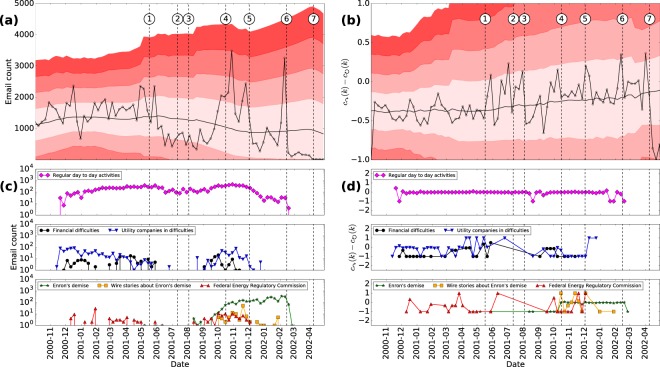
Time series of Enron events. (**a**) Time series of the number of emails. (**b**) Time series of the difference between the inter- and intra-community link ratios. (**c**) Time series of the number of emails classified by topics. (**d**) Time series of the difference between the inter- and intra-community link ratio classified by topics.

Using the email data from Enron, we compare the proposed method against the baseline of email volume. Figure 2 shows the time series of the volume of emails sent between 2000-09-30 and 2002-04-30. First, we consider whether the volume of emails correlates with the events associated to Enron’s collapse (depicted by the dashed vertical lines). Figure 2(a) shows the weekly volume of emails. The horizontal solid line represents the moving average of emails during the observation period using a window length equivalent to a year of data (52 weeks). Each horizontal red band represents one moving standard deviation from the moving average using the same window length (more intense bands indicate observations that are further away from the mean, based on Algorithm 1). Note that events 1, 4, 5 and 6 lie more than one standard deviation away from the moving average and their occurrence coincides with a burst of emails. However, this relationship does not hold for events 2, 3 and 7.

We measure the difference between inter- and intra-community link ratios detailed in equations (1) and (2). For the Enron dataset, the community partition results from a period of ${m}_{0}=91$ weeks. For discussion on selecting the appropriate value of *m*_0_, see Supplementary Information (SI), Section S1. Figure 2(b) shows that the occurrence of events, 1 through 7, coincide with peaks in the proposed measure. Even events that do not occur during periods of elevated volume of emails are associated with increased inter-community communication. This result supports our hypothesis that there is a considerable transmission of information through inter-community links when events take place. Note that the activity signal may occur before or after the events. It is natural to expect heightened activity before the “event” in many cases. The “events” in the Enron’s case are the public release of certain information. Therefore, it is reasonable to assume that, in some cases, such information had been circulating internally, preceding the actual “event.”

Figure 2(c) shows the volume classified into six topics^24^. One topic represents contents associated to daily activities; the remaining ones are associated to Enron’s bankruptcy. Most emails are classified into day-to-day activities. For the categories not related to Enron’s bankruptcy, there is no association between events and topics, suggesting that the volume of emails does not help us to characterize a detection pattern.

Figure 2(d) shows the difference between inter- and intra-community link ratios distinguished by topics. Note the association between events and topics. In particular, emails about day-to-day activities have a similar inter- and intra-community diffusion pattern during the entire observation period, depicted by the flat curve. For topics related to “utility companies difficulties,” “Federal Energy Regulatory Commission” and “wire stories about Enron’s demise,” there is a positive association. In other words, topics that are sensitive to bankruptcy diffuse across communities. The co-occurrence of peaks and events in Fig. 2(b) shows that the proposed criterion can be used as a signature for detection.

**Figure 3:**
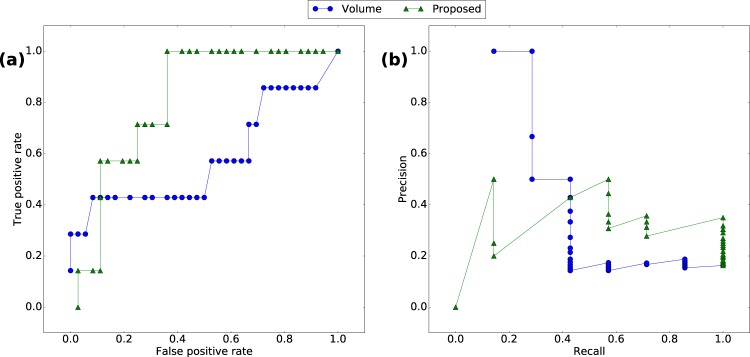
Performance comparison for the Enron case when $m=2$ weeks. (**a**) ROC. (**b**) PRC.

**Figure 4:**
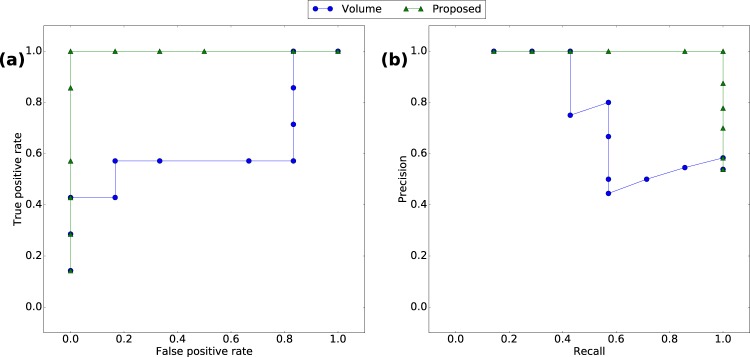
Performance comparison for the Enron case when $m=7$ weeks. (**a**) ROC. (**b**) PRC.

Figures 3 and 4 show the performance of detecting events using the proposed criterion against the volume of emails (for different detection resolutions). The detection resolution, denoted by *m*, describes the number of weeks within which we compute the output of the detection algorithm. We compare detection algorithms using the ROC^25^ and the PRC^26^ to take into account that the dataset is unbalanced^27^. The proposed approach performs generally better than volume-based detection, with noticeable improvements at lower resolutions. For $m=7$, the proposed method has a perfect performance.

### Boston marathon

#### Mention network

**Figure 5:**
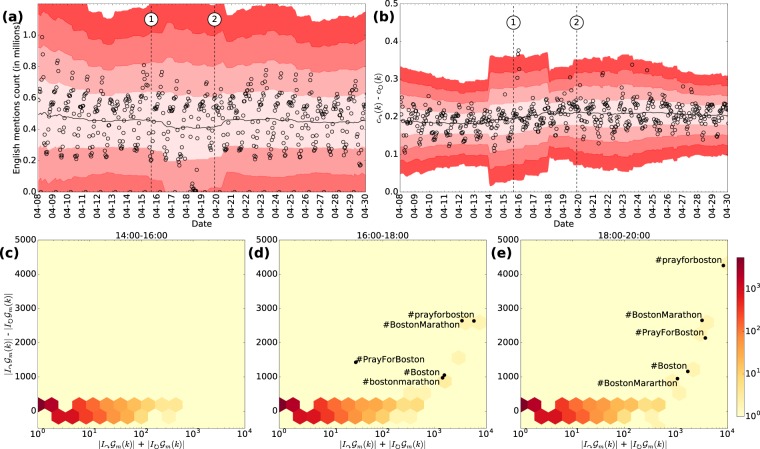
Time series analysis of the mention network. (**a**) Time series of the number of mentions. (**b**) Time series of the difference between the inter- and intra-community link ratios. (**c**) Distribution of the number of hashtags based on the total number of links (horizontal axis) and the difference of inter- and intra-community links (vertical axis) during the interval 14:00-16:00 EST on 2013-04-15. (**d**) Same as (**c**) during the interval 16:00-18:00 EST. (**e**) Same as (**c**) during the interval 18:00-20:00 EST. Hashtags related to the Boston Marathon bombing are highlighted.

As in the previous section, we evaluate whether the communication volume relates to events associated with the Boston Marathon bombing (depicted by the dashed vertical lines and numbers). Figure 5(a) shows the hourly number of English mentions during April 2013. We use the same visualization conventions as in Fig. 2(a,b). Here the window length of the moving average is equivalent to four days of data (96 samples). Note that on 2013-04-08 at 07:00 UTC, there are some observations that fall three standard deviations beyond the mean. However, these observations are not associated with the events of interest. Similarly, on 2013-04-18, there is a significant decrease in the number of mentions due to missing data. Note also that around events 1 and 2 the number of mentions is comparable to the one in other hours during the observation period, i.e., these data points are statistically insignificant.

Figure 5(b) shows the difference between inter- and intra-community link ratios which demonstrates significant deviation at the time of the events (for ${m}_{0}=7$ days). For event 1, the difference moves beyond four standard deviations—suggesting a significant increased in inter- compared to intra-community communications. For event 2, the difference between the ratios is three standard deviations, which is still noticeable compared to other times during the observation period. Figure 5(b) also shows other significant deviations. In particular, on 2013-04-21 at 17:00 UTC and 18:00 UTC, the proposed measure falls three standard deviations from the average. This behavior coincides with the hacking of the Associated Press Twitter account on 2013-04-21 around 17:00 UTC. A fake message reported that there had been “two explosions in the white house and Barack Obama [was] injured,” which caused financial markets to panic for a few minutes^28^.

We also analyze the contents of the mentions. Figure 5(c–e) show the distribution of the activity of each hashtag mentioned on 2013-04-15 at different time intervals after the bombing (in EST). In particular, we measure the total number of communications and the difference of the two modalities of communication (inter- and intra communication links). The red cells around the origin indicate that most hashtags are not frequently used. Note also that hashtags tend to be confined inside communities. This is evidenced by the absence of observations with large difference between inter- and intra-communication. Right after the bombing (at 15:00 EST) there are no hashtags with significant difference in the inter- and intra-community level in Fig. 5(c). However, in the two subsequent two hour intervals, hashtags related to the bombing emerged distinguished by lots of inter-community communications (see Fig. 5(d,e)). The highlighted cells correspond to the bombing related hashtags #prayforboston, #BostonMarathon, #PrayForBoston, #Boston and #BostonMararthon. These results demonstrate that the increase in the difference between inter- and intra-community communication is indeed triggered and driven by the bombing event.

#### Retweet network

**Figure 6:**
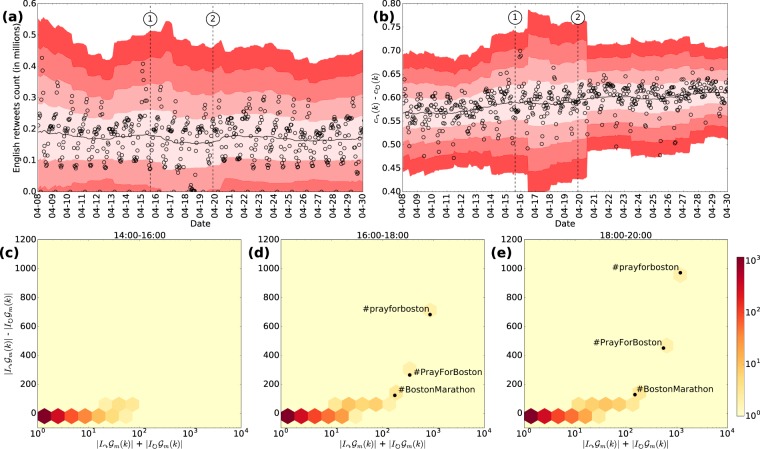
Time series analysis of the retweet network. We perform the same analysis as for the mention network and report the number of retweets in (**a**), the difference between inter- and intra-community link ratios in (**b**), the distribution of the number of hashtags based on the total number of links (horizontal axis); and the difference of inter- and intra-community links (vertical axis) during the interval 14:00-16:00 EST on 2013-04-15 in (**c**). (**d**) Same as (**c**) during the interval 16:00-18:00 EST. (**e**) Same as (**c**) during the interval 18:00-20:00 EST. Hashtags related to the Boston Marathon bombing are highlighted.

Figure 6(a) shows the hourly number of English retweets. Note that on 2013-04-08 at 07:00 UTC, there are observations that lay up to three standard deviations from the mean, but are not associated with the events of interest. The spike in the retweet activity is *before* the bombing, which does not relate to the bombing event.

Figure 6(b) shows the difference between inter- and intra-community link ratios (${m}_{0}=7$ days). For event 1, the difference between community ratios spiked up to four standard deviations, in accordance with Fig. 5(b). For event 2, the difference between community ratios is not as significant as for the case of mentions in Fig. 5(b). Other relevant deviations are not observed during the period.

We also explore the content diffused in the retweet network during the hours of the bombing. We measure the distribution of the number of links for each hashtag retweeted on 2013-04-15 at the same time intervals used for Fig. 5(c–e) and reported them in Fig. 6(c–e). For the retweet network, we do not observe a significant distribution of hashtags in the vertical axis after the bombing event (see Fig. 6(c)). However, Figs 5(d,e) and 6(d,e) show that the bombing related hashtags are placed in regions of low density but with significant difference in inter- and intra-communication links. These hashtags correspond to #prayforboston, #PrayForBoston and #BostonMarathon.

## Discussion

This paper demonstrates a proof-of-concept of a community-based method to detect the occurrence of global events in temporal networks. In doing so, we put forward a novel, theoretically grounded approach toward systematic event detection. We apply the proposed method to (*i*) the Enron email dataset (during its collapse period); and (*ii*) the Twitter mention and retweet dataset (when the Boston Marathon bombing took place). We hypothesize that events like Enron’s bankruptcy and the bombing (along with the manhunt) are relevant to many people regardless of their regular community membership in communication networks. These events prompt more communication across community boundaries (there is increased communication between users of different communities). As a consequence of the occurrence of global events, communication patterns become more diverse.

Our work exhibits the following limitations. First, the proposed method depends on the definition of an initial community partition. The initial community partition is defined by aggregating the network activity during a fixed period of time (i.e., controlled through *m*_0_). By relying on this strategy, we guarantee that the majority of the users are going to be identified with a community partition and that subsequent interactions in the communication networks can be classified with respect to inter- or intra-community links. However, there is a lot of freedom on the strategy to prepare and update this “normal” community structure. Second, to make the decision on whether a particular data sample includes an event, our reasoning is based on the distance of the observation with respect the moving average of the measure. We implement this criterion by accounting for the number of moving standard deviations (i.e., controlled through *δ* in Algorithm 1). Clearly, defining how many standard deviations are needed to establish a detection threshold depends on many factors. In the two case studies, the frequency of the formation of the networks is one week for Enron and one hour for the Twitter datasets. Third, in evaluating the performance of the algorithms, the detection intervals are assumed to be proportional to the network formation intervals. This means that even when an interval is reported to contain an event, there is no notion of temporality with respect to the closeness of the occurrence of the event within that interval. This might be balanced by increasing the length of detection intervals. However, given the length of the observation periods, a limited detection resolution will decrease the performance of the proposed method.

## Methods

### Data

#### Enron email communication network

Enron was one of the largest U.S. businesses in the late 90s when it filed for bankruptcy in 2001^21^. The company omitted negative balances and reported inflated profits by allocating losses into fraudulent special purpose entities. After its investigation, the Federal Regulatory Commission published a corpus of Enron’s corporate emails^29,30^, consisting of over 125000 emails sent by 184 employees. The data can be represented as a directed weighted network in which a node is an employee and a link is the number of emails between two employees. The network describes interactions between 1999-01-01 and 2002-04-30. Across the email exchange that led to the bankruptcy, seven major events have been identified^31,32^. The events are described in Table 1.

**Table 1:** Enron’s event description.

Event ID	Date	Description
1	2001-05-17	Schwarzenegger, Lay, Milken meeting.
2	2001-07-12	Quarterly conference call.
3	2001-08-03	Skilling makes a bullish speech on Enron Energy Services. That afternoon, he lays off 300 employees.
4	2001-10-16	Enron reports a 618 million third-quarter loss and declares a 1.01 billion non-recurring charge against its balance sheet, partly related to “structured finance” operations run by chief financial officer Andrew Fastow. In the analyst conference call that day, Lay also announces a 1.2 billion cut in shareholder equity.
5	2001-12-02	Enron, at the time the largest bankruptcy in U.S. history, files for Chapter 11 bankruptcy protection.
6	2002-02-14	Sherron Watkins, the Enron whistleblower, testifies before a Congressional panel against Skilling and Lay.
7	2002-04-09	David Duncan, Arthur Andersen’s former top auditor, pleads guilty to obstruction.

#### Twitter interaction networks during the Boston Marathon bombing

On April 15th 2013, explosions took place during the Boston Marathon^33^. One of two suspects was shot dead on April 18th and the other was captured on April 19th^34^. We use over 456 million English tweets, posted during April, to create a mention and a retweet network. The events that we consider are described in Table 2 and have been referenced in previous studies^33,34^.

**Table 2:** Boston Marathon bombing event description.

Event ID	Date	Time	Description
1	2013-04-15	14:49 (UTC)	Bombing
2	2013-04-19	20:42 (UTC)	Manhunt

### Network representation

Consider the sequence of *n* equal-sized intervals $A=\{{A}_{1},{A}_{2},\ldots ,{A}_{n}\}={\{{A}_{k}\}}_{k=1}^{n}$. Let ${\mathcal H} =\{1,2,\ldots ,N\}$ be the set of nodes (e.g., the set of Enron employees or Twitter users). Let ${\mathscr{V}}(k)\subseteq  {\mathcal H}$ be the subset of nodes that interact during interval ${A}_{k}=[{a}_{k},{a^{\prime} }_{k})$. Let ${\mathscr{W}}(k)=\{{\omega }_{ij}(k):i,j\in {\mathscr{V}}(k)\}$ be a weighted adjacency matrix in which ${\omega }_{ij}(k)$ captures the number of interactions between nodes *i* and *j*. Let ${\mathscr{G}}(k)=({\mathscr{V}}(k),{\mathscr{W}}(k))$ represent a weighted directed network that takes account of all interactions within interval *A*_*k*_. Finally, let $G={\{{\mathscr{G}}(k)\}}_{k=1}^{n}$ denote the sequence of the temporal networks.

#### Detection problem

The series *G* captures the dynamics of the network across time and defines the basis for detection. Let *m* ($1\le m < n$) represents the resolution of detection in terms of the number of intervals ${A}_{k},k\in \{1,2,\ldots ,n\}$. For instance, if $m=2$, then the detection problem is concerned with identifying whether an event occurs within the detection interval $({a}_{k-m+1},{a^{\prime} }_{k}]=({a}_{k-1},{a^{\prime} }_{k}]$, $k=1,2,\ldots n$. Let $\bar{n}=\lfloor \frac{n}{m}\rfloor$ be the number of times an algorithm (with resolution *m*) assesses detection. Let $E\subseteq \{1,2,\ldots ,\bar{n}\}$ represent the intervals at which at least one event occurs (based on ground truth information). Define $e\in E$ as the index of a detection interval containing an event. Let $\hat{E}\subseteq \{1,2,\ldots ,\bar{n}\}$ represent the set of intervals at which the occurrence of at least one event is reported by the detection method. Similarly $\hat{e}\in \hat{E}$ represents the index at which an event is reported. The detection problem is defined as follows: Given a series of networks $G={\{{\mathscr{G}}(k)\}}_{k=1}^{n}$ and a detection resolution *m*, identify the set of intervals $\hat{E}$ that contain at least one event.

### Method evaluation

The performance of the algorithm is measured based on $\hat{E}$ and *E*, with which we calculate ROC and PRC using the values of the generated time series as a threshold.

### The proposed detection method

The proposed method identifies detection signatures based on the communication patterns with respect to a network community and not in the change of the community structure. Thus, rather than trying to detect community structure in each time step, we aggregate network snapshots to build an initial network segment at which we apply community detection and consider the resulting partition as the normal community. For each time period, the network snapshot is defined by the communication occurred during the time period.

The initial network segment of length *m*_0_ is defined by

$${{\mathscr{G}}}_{{m}_{0}}=({{\mathscr{V}}}_{{m}_{0}},{{\mathscr{W}}}_{{m}_{0}})=\underset{k^{\prime} =1}{\overset{{m}_{0}}{\bigoplus }}\,{\mathscr{G}}(k^{\prime} )={\mathscr{G}}(1)\oplus \cdots \oplus {\mathscr{G}}({m}_{0})$$

where ${{\mathscr{V}}}_{{m}_{0}}={\cup }_{k^{\prime} =1}^{{m}_{0}}\,{\mathscr{V}}(k^{\prime} )$ and ${{\mathscr{W}}}_{{m}_{0}}={\sum }_{k^{\prime} =1}^{{m}_{0}}\,{\mathscr{W}}(k^{\prime} )$. In this network, we consider only reciprocal communications within the largest connected component. We also remove dangling nodes. From the initial network segment ${{\mathscr{G}}}_{{m}_{0}}$, we identify non-overlapping communities, i.e., the set of nodes can be grouped into subsets such that nodes belonging to the same subset are densely interconnected^35^.

The proposed algorithm reports events based on the proportion of inter- and intra-community links of the network ${{\mathscr{G}}}_{m}(k)$ with respect to ${m}_{0}$. For the Enron network, the community partition used as a reference corresponds to a period of ${m}_{0}=91$ weeks. In this network, ${{\mathscr{G}}}_{{m}_{0}}$ has 222 nodes and 28672 edges before simplification. After simplification, ${{\mathscr{G}}}_{{m}_{0}}$ has 81 nodes and 199 edges. We identify eight communities. For the Twitter networks, *m*_0_ corresponds to seven days of user interactions. For the mention network, ${{\mathscr{G}}}_{{m}_{0}}$ has 20597742 nodes and 60147176 edges before simplification. After simplification, ${{\mathscr{G}}}_{{m}_{0}}$ has 3517744 nodes and 3626560 edges. We identify 665656 communities. For the retweet network, ${{\mathscr{G}}}_{{m}_{0}}$ has 12209899 nodes and 26215122 edges before simplification. After simplification, ${{\mathscr{G}}}_{{m}_{0}}$ has 588279 nodes and 433583 edges. We identify 181007 communities.

To define $\hat{E}$, let $C({{\mathscr{G}}}_{{m}_{0}})=\{0,1,\ldots ,c\}$ be a set of unique community identifiers, where $c+1$ is the number of communities in ${{\mathscr{G}}}_{{m}_{0}}$. The community to which node $i\in {{\mathscr{V}}}_{m}(k)\cap {{\mathscr{V}}}_{{m}_{0}}$ belongs (based on ${{\mathscr{G}}}_{{m}_{0}}$) is given by ${c}_{i}:i\to C({{\mathscr{G}}}_{{m}_{0}})$. We compute the community partition of ${{\mathscr{G}}}_{{m}_{0}}$ using the Infomap algorithm^36^. Following similar ideas as in^20^, let ${I}_{\curvearrowright }({{\mathscr{G}}}_{m}(k))=\{(i,j):{\omega }_{ij}(k) > 0\wedge ({c}_{i}\cap {c}_{j})=\varnothing \}$ represent the set on inter-community links and ${I}_{\circlearrowright }({{\mathscr{G}}}_{m}(k))=\{(i,j):{\omega }_{ij}(k) > 0\wedge ({c}_{i}\cap {c}_{j})\ne \varnothing \}$ the set of intra-community links. Define the inter- and intra-community link ratios as

1 $${c}_{\curvearrowright }^{m}(k)=\frac{|{I}_{\curvearrowright }({{\mathscr{G}}}_{m}(k)|}{|{I}_{\curvearrowright }({{\mathscr{G}}}_{m}(k))|+|{I}_{\circlearrowright }({{\mathscr{G}}}_{m}(k))|}$$

2 $${c}_{\circlearrowright }^{m}(k)=\frac{|{I}_{\circlearrowright }({{\mathscr{G}}}_{m}(k)|}{|{I}_{\curvearrowright }({{\mathscr{G}}}_{m}(k))|+|{I}_{\circlearrowright }({{\mathscr{G}}}_{m}(k))|}$$

Detection focuses on identifying the intervals *k*, for which ${c}_{\curvearrowright }^{m}(k)-{c}_{\circlearrowright }^{m}(k)$ exceeds a threshold that is a function of the mean and the standard deviation. We use the sample mean (over the entire period of the study) as the mean estimator because observations seem to resemble a normal distribution—since hypothesis testing demonstrates that the normal distribution is a good candidate to model the generation of the empirical observations. Moreover, we use the sample standard deviation as the estimator of the standard deviation. The pseudo-code for the detection algorithm is shown in Algorithm 1. The parameter *δ* controls how many standard deviations are considered to report an event. The parameter *τ* is the window length in the moving average model.

**Algorithm 1:**
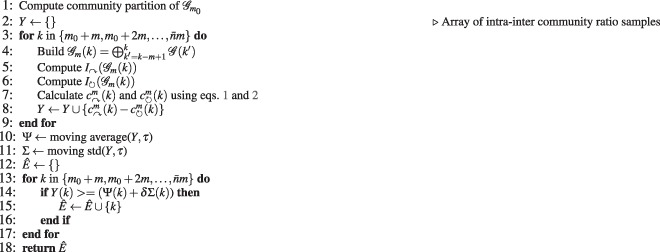
Event-Detection (*G*, *m*_0_, *m*, *δ*, *τ*).

To measure detection performance, we compare the measure of ${c}_{\curvearrowright }^{m}(k)-{c}_{\circlearrowright }^{m}(k)$ with the respective measure derived from ${{\mathscr{G}}}_{m}(k)$, e.g., the volume of interactions—number of links of the cumulative network segment.

## Supplementary information

supplement-doc.pdf

## Data Availability

The datasets analyzed during the current study are available at http://www.cis.jhu.edu/~parky/Enron/ (Enron) and 10.5281/zenodo.1321085 (Twitter). The code for the proposed method is available at https://github.com/pmoriano/Community-Based-Event-Detection and released under the GNU General Public License.
