# Supplementary material for: Community-Based Event Detection in Temporal Networks
Source: Sci Rep. 2019 Mar 13;9:4358. doi: 10.1038/s41598-019-40137-0 (PMC6416296; doi:10.1038/s41598-019-40137-0)
Supplement: Supplementary file 1 — supplement-doc.pdf [file 41598_2019_40137_MOESM1_ESM.pdf]

Community-Based Event Detection in Temporal Networks
Supplementary Information

Pablo Moriano*[1,2], Jorge Finke[3], and Yong-Yeol Ahn[1]

[1]Center for Complex Networks and Systems Research, School of Informatics, Computing, and Engineering, Indiana University, Bloomington, IN 47408, USA
[2]Center for Security and Privacy in Informatics, Computing, and Engineering, School of Informatics, Computing, and Engineering, Indiana University, Bloomington, IN 47408, USA
[3]Department of Electrical Engineering and Computer Science, Pontificia Universidad Javeriana, Cali, Colombia

S1. Selection of m_0

The selection of m_0 is an important step of the proposed method. We aggregated weekly email data from 1999-01-01 until 2001-05-29 (which coincides with the date of the first event). For each of these aggregated networks, we computed common network properties shown in Fig. 1. The dashed line represents the date that we used to define m_0, i.e., 2000-10-01. Note that for the selection of m_0, the network density and modularity reached stationary values.

Figure 2 shows the difference between inter- and intra-community link ratios for different values of m_0 based on aggregated networks from 2000-09-15 until 2000-10-14. Fig. 3 shows the classification results based on ROC for the same values of m_0 when $m = 2$. For this value of m, the classification results tend to be better when m_0 is around 91 weeks (i.e., reached in 2000-10-01). Note that the peaks on the proposed measure are closely aligned with the dates of the events for different values of m_0. Figure 4 shows a similar effect for classification results based on PRC. However, PRC results are sensible to changes in m_0. We also explored the effect of m_0 on the detection performance when $m = 7$. Figure 5 shows the results based on ROC. In contrast to Fig. 3, the results in this case are less sensible to m_0. They start to distort for values of m_0 above 92 weeks (i.e., after 2000-10-04). These results are consistent with performance changes observed in the classification results based on PRC in Fig. 6.

*pmoriano@indiana.edu. Corresponding author.

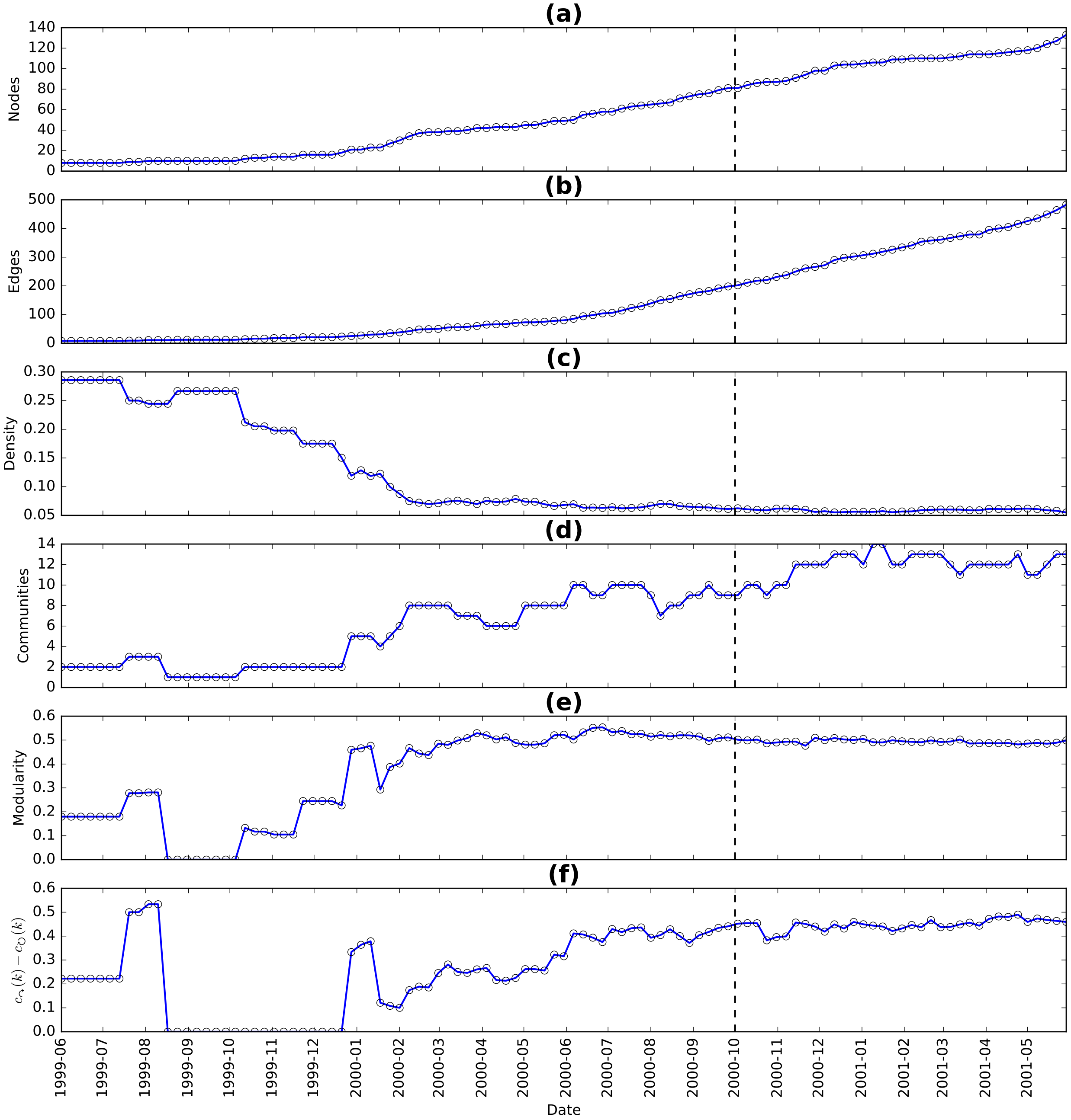

Figure 1: **Time series of network properties for the cumulative graph segment $\mathcal{G}_{m_0}$.** (**a**) Number of nodes. (**b**) Number of edges. (**c**) Density of the network. (**d**) Number of communities. (**e**) Modularity. (**f**) Difference between inter- and intra-communications. Dashed lines indicated the final date used to generate the aggregated network $\mathcal{G}_0$ for the Enron dataset (i.e., 2000-10-01).

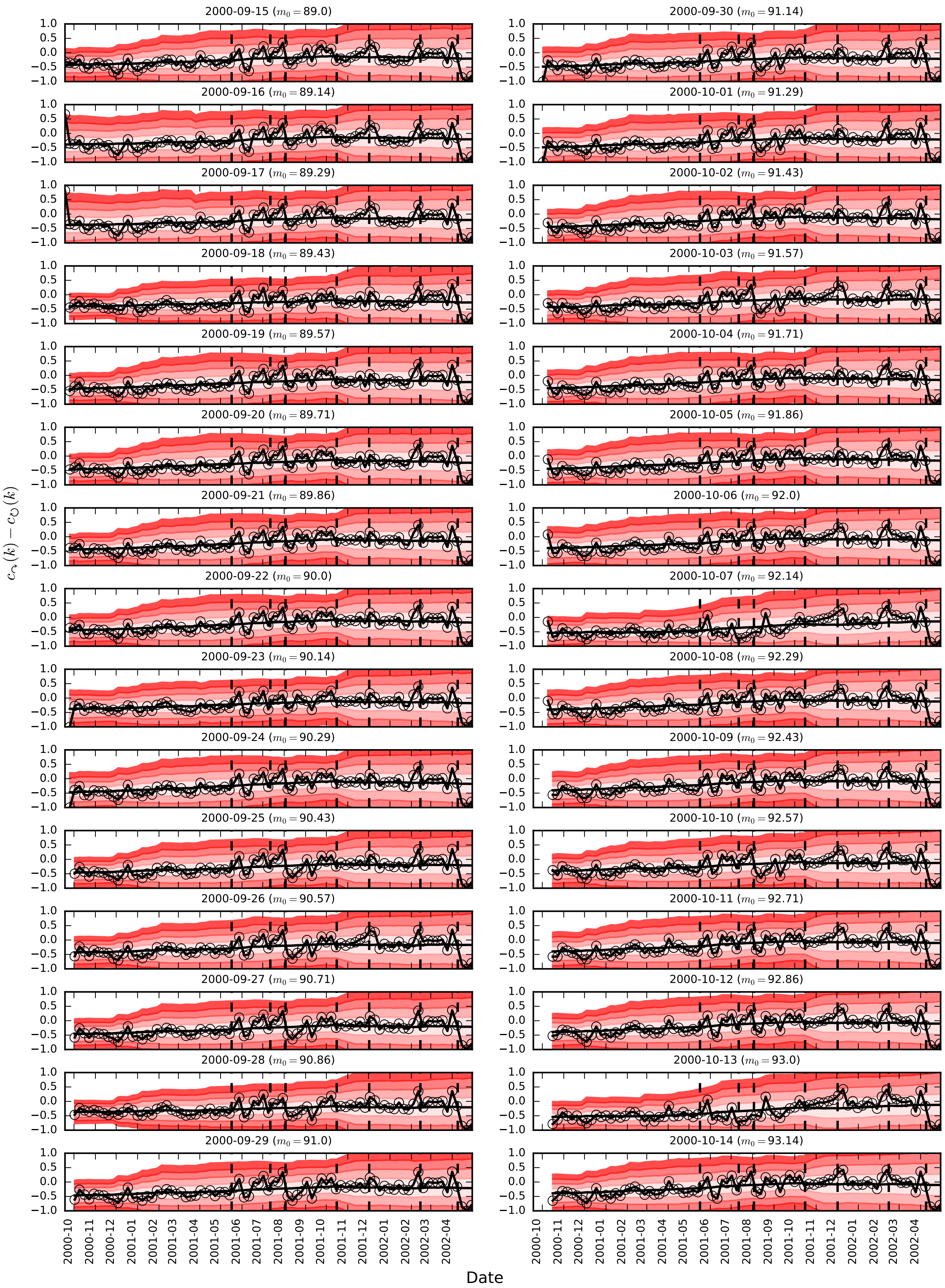

Figure 2: **Time series of the difference between inter- and intra-community links ratios for different values of m_0.** Dashed lines correspond to the events reported for the Enron dataset.

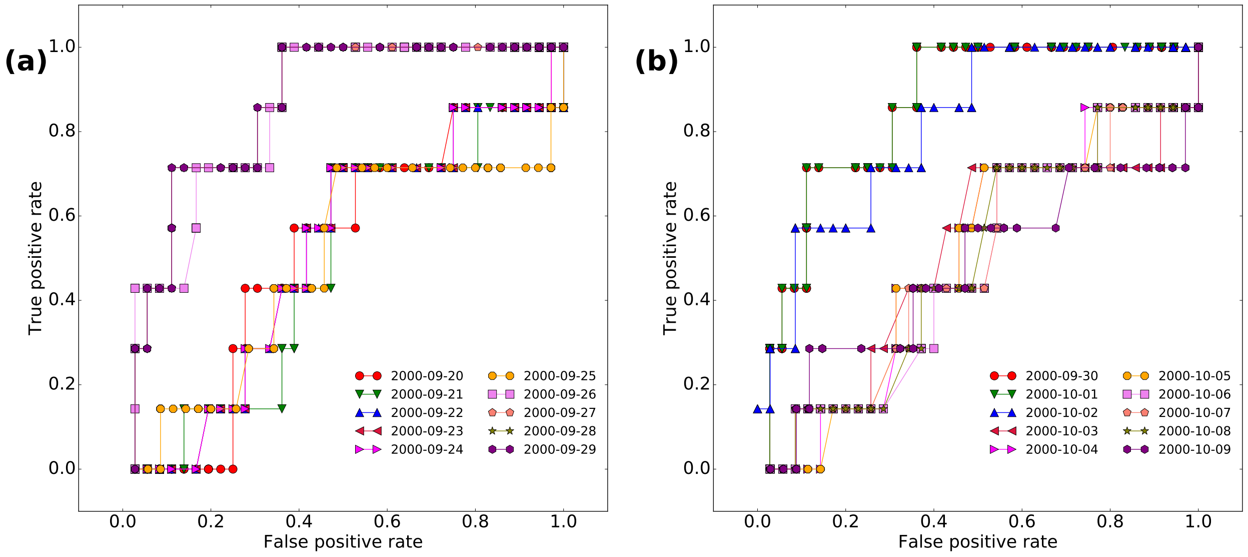

Figure 3: **Performance comparison using ROC for $m = 2$ and different values of m_0.** (**a**) For m_0 representing aggregate networks with final date between 2000-09-20 and 2000-09-29. (**b**) For m_0 representing aggregate networks with final date between 2000-09-30 and 2000-10-09.

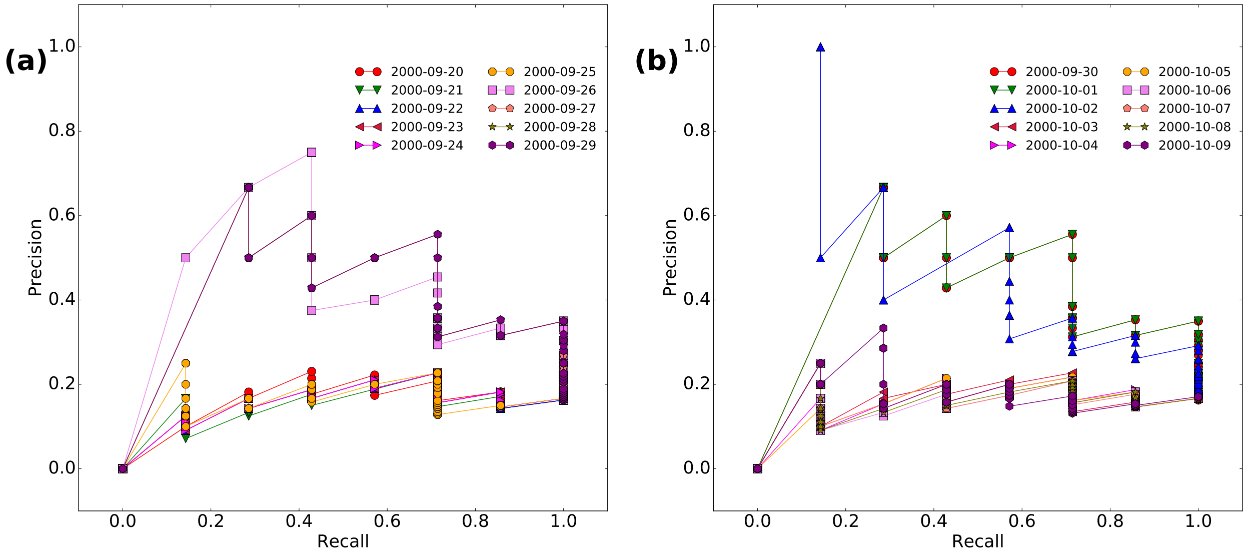

Figure 4: **Performance comparison using PRC for $m = 2$ and different values of m_0.** (**a**) For m_0 representing aggregate networks with final date between 2000-09-20 and 2000-09-29. (**b**) For m_0 representing aggregate networks with final date between 2000-09-30 and 2000-10-09.

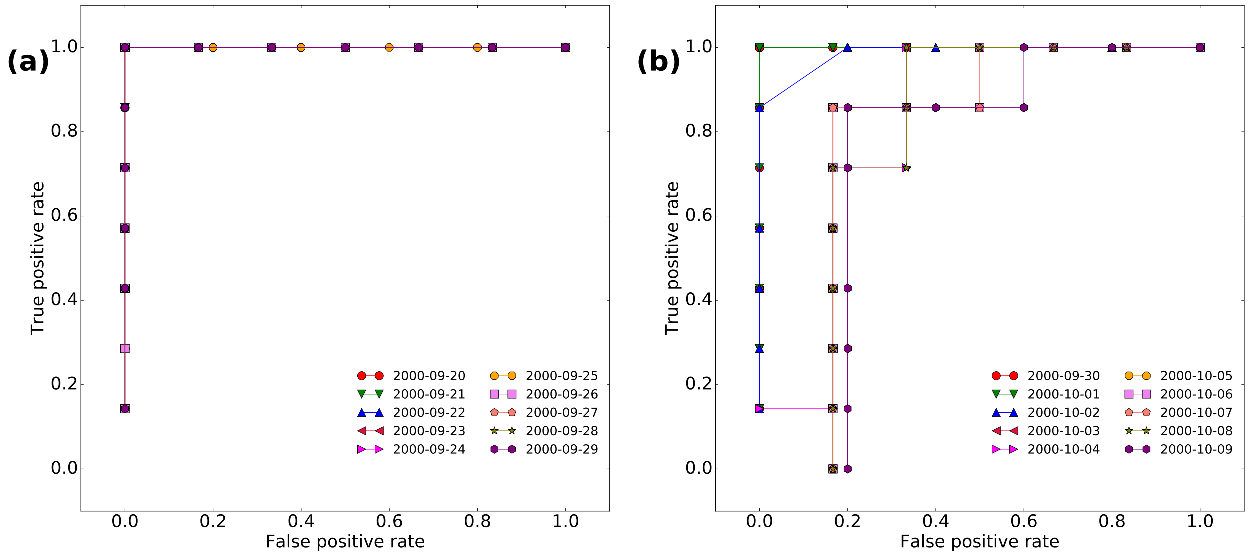

Figure 5: **Performance comparison using ROC for $m = 7$ and different values of m_0.** (**a**) For m_0 representing aggregate networks with final date between 2000-09-20 and 2000-09-29. (**b**) For m_0 representing aggregate networks with final date between 2000-09-30 and 2000-10-09.

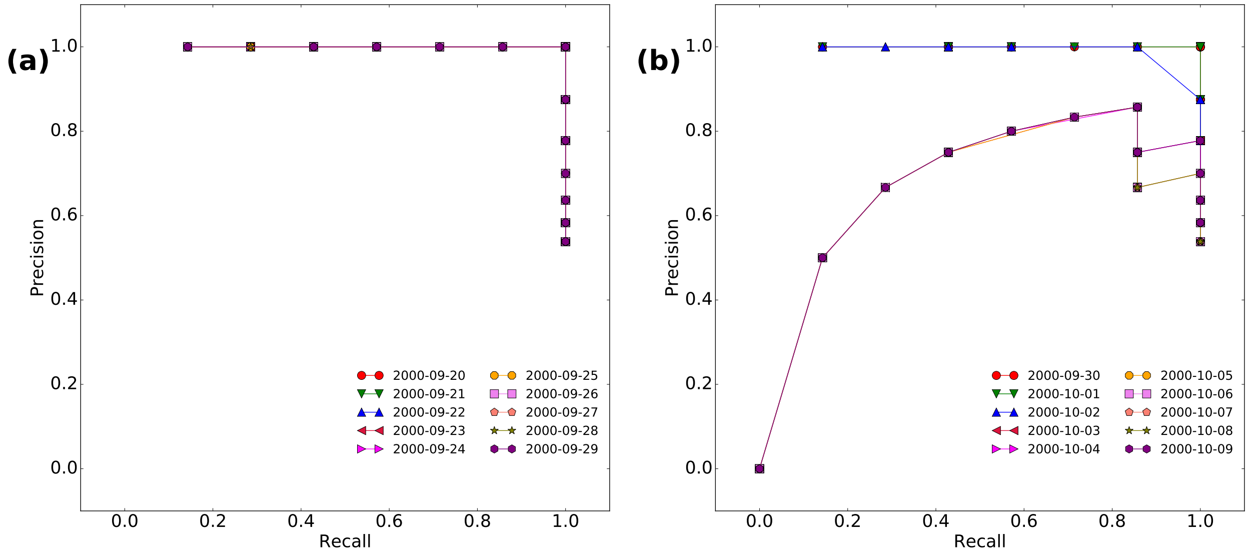

Figure 6: **Performance comparison using PRC for $m = 7$ and different values of m_0.** (**a**) For m_0 representing aggregate networks with final date between 2000-09-20 and 2000-09-29. (**b**) For m_0 representing aggregate networks with final date between 2000-09-30 and 2000-10-09.
